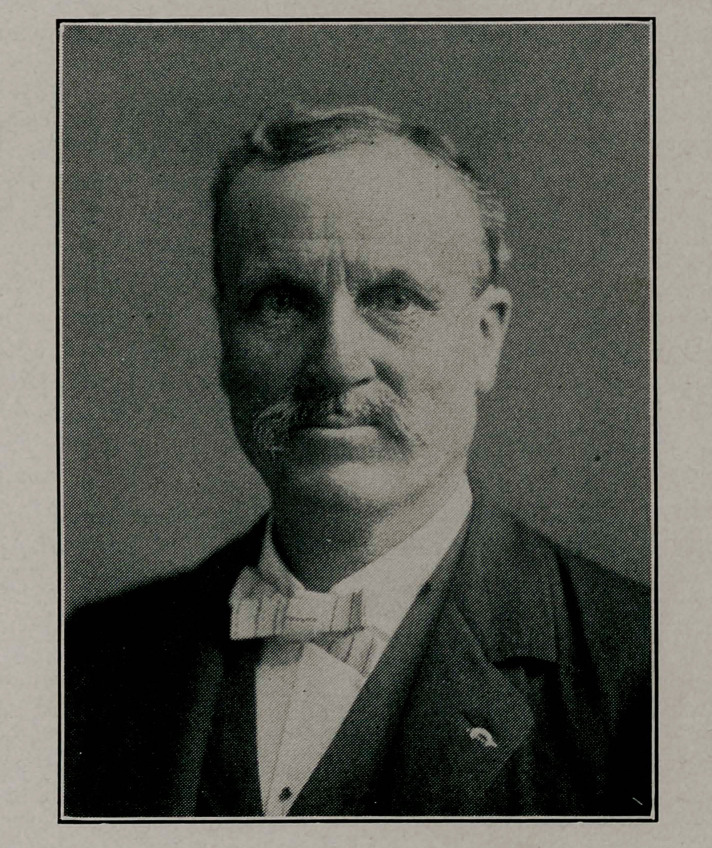# Dr. James Sadequist Smith

**Published:** 1912-03

**Authors:** 


### OBITUARY

"Dr. James Sadequist Smith, who died January 30th, 1912, was born in Smithtown, near St. John's, New Brunswick on December 6th 1831. After receiving his degree at the University of Buffalo, 1852, he went into partnership with Dr. Milton Potter, and for a number of years was on the obstetric staff of St. Mary's Infant Asylum on Edward Street. With the exception of this obstetric work, he devoted his time to general practice.

About 1870, he contracted pulmonary tuberculosis and, for a number of years, was considered to be in a dangerous condition, but, thanks to a natural vitality and an out of door life, he recovered his health and resumed his practice.

He was a member of the Erie County Medical Society, Academy of Medicine, Queen City Lodge of Masons and Commandery.

He served as Assistant Surgeon and Surgeon of the 65th Regiment, and as 4th Brigade Surgeon with rank as Major, and brought up his son, Lt. Col. Eugene A. Smith, M. D., with a love of military service and rifle shooting.

Dr. Smith built up a large practice and was a busy man, but was never too busy to stop and chat with his friends for whom he always had a smile. He was courteous and affable to all, and will be long remembered and missed among his patients and medical associates."

OUR thanks are due to the *Buffalo Express* for the use of the picture of the late Dr. Smith.

---

OUR readers are requested to send notices of deaths of physicians in western New York, or who have resided elsewhere but have been graduates of schools of this region or who have otherwise been identified with this region. State residence, date of death, age, cause of death,school of graduation, and other items of interest.

## Figures and Tables

**Figure f1:**